# Landsat-based spatiotemporal estimation of subtropical forest aboveground carbon storage using machine learning algorithms with hyperparameter tuning

**DOI:** 10.3389/fpls.2024.1421567

**Published:** 2024-08-29

**Authors:** Lei Huang, Zihao Huang, Weilong Zhou, Sumei Wu, Xuejian Li, Fangjie Mao, Meixuan Song, Yinyin Zhao, Lujin Lv, Jiacong Yu, Huaqiang Du

**Affiliations:** ^1^ State Key Laboratory of Subtropical Silviculture, Zhejiang A & F University, Hangzhou, China; ^2^ Key Laboratory of Carbon Cycling in Forest Ecosystems and Carbon Sequestration of Zhejiang Province, Zhejiang A & F University, Hangzhou, China; ^3^ Qianjiangyuan-Baishanzu National Park, Lishui, Zhejiang, China

**Keywords:** subtropical forest, AGC, machine learning, remote sensing, Lishui City

## Abstract

**Introduction:**

The aboveground carbon storage (AGC) in forests serves as a crucial metric for evaluating both the composition of the forest ecosystem and the quality of the forest. It also plays a significant role in assessing the quality of regional ecosystems. However, current technical limitations introduce a degree of uncertainty in estimating forest AGC at a regional scale. Despite these challenges, remote sensing technology provides an accurate means of monitoring forest AGC. Furthermore, the implementation of machine learning algorithms can enhance the precision of AGC estimates. Lishui City, with its rich forest resources and an approximate forest coverage rate of 80%, serves as a representative example of the typical subtropical forest distribution in Zhejiang Province.

**Methods:**

Therefore, this study uses Landsat remote sensing images, employing backpropagation neural network (BPNN), random forest (RF), and categorical boosting (CatBoost) to model the forest AGC of Lishui City, selecting the best model to estimate and analyze its forest AGC spatiotemporal dynamics over the past 30 years (1989–2019).

**Results:**

The study shows that: (1) The texture information calculated based on 9×9 and 11×11 windows is an important variable in constructing the remote sensing estimation model of the forest AGC in Lishui City; (2) All three machine learning techniques are capable of estimating forest AGC in Lishui City with high precision. Notably, the CatBoost algorithm outperforms the others in terms of accuracy, achieving a model training accuracy and testing accuracy R^2^ of 0.95 and 0.83, and RMSE of 2.98 Mg C ha^-1^ and 4.93 Mg C ha^-1^, respectively. (3) Spatially, the central and southwestern regions of Lishui City exhibit high levels of forest AGC, whereas the eastern and northeastern regions display comparatively lower levels. Over time, there has been a consistent increase in the total forest AGC in Lishui City over the past three decades, escalating from 1.36×10^7^ Mg C in 1989 to 6.16×10^7^ Mg C in 2019.

**Discussion:**

This study provided a set of effective hyperparameters and model of machine learning suitable for subtropical forests and a reference data for improving carbon sequestration capacity of subtropical forests in Lishui City.

## 1 Introduction

The aboveground carbon storage (AGC) in forests is one of the important indicators for evaluating the structure of forest ecosystems and the quality of forests. It also serves as a significant measure for evaluating the quality of the regional ecological environment (Diao et al., 2022). Accurately quantifying forest carbon storage and monitoring its spatial distribution is beneficial for a more concrete understanding of the terrestrial carbon cycle process and understanding the carbon sink patterns in different regions. It also allows for a more accurate assessment of the potential of forest carbon sinks. This has significant implications for the formulation of carbon sequestration and emission reduction policies (Liu and Wu, 2017; Su et al., 2016).

Currently, the estimation of forest AGC mainly falls into three methods: field survey, model simulation, and remote sensing estimation (Gomez and Gumersindo, 2017; Mngadi et al., 2021). The traditional field survey method is the most intuitive and accurate, but it is difficult to reflect the situation of the entire forest area. This approach necessitates substantial human and material resources and is inherently damaging. Model simulation methods such as Biome-BioGeochemical Cycles (BIOME-BGC) require a variety of vegetation parameters to estimate the forest AGC. A lack of sufficient input data or the presence of missing data can notably influence the accuracy of the prediction outcomes (Liu et al., 2019). With the gradual development of forest AGC estimation research technology and the continuous optimization of research methods, remote sensing estimation methods have gradually replaced field survey methods as the main research methods for estimating forest AGC (Stovall et al., 2017). This method infers and estimates the carbon storage on the Earth’s surface by acquiring remote sensing data of the Earth’s surface and combining it with ground monitoring data and model algorithms (Li et al., 2022). Since its launch, the Landsat series of satellites has demonstrated advantages such as a good performance-price ratio, rich spectral information, and a quick image update cycle. As a result, Landsat has become the most widely used remote sensing data source in many applications, including estimation of AGC, land use/cover surveys, agricultural yield estimation, regional planning, and forest fire monitoring (Wang and Gao, 2019; Li H, et al., 2023; Lu, 2006; Xu et al., 2011; Du et al., 2008; Li et al., 2018).

However, remote sensing data cannot directly reveal the forest AGC and its changes. It is necessary to establish a complete mathematical model between the information received by the satellite and the ground-measured AGC to realize the spatiotemporal estimation of forest AGC (Du et al., 2012; Zhang et al., 2019). Among the many AGC estimation models, machine learning algorithms such as backpropagation neural network (BPNN), support vector regression (SVR), random forest (RF), and ensemble learning (EL) are widely used for forest AGC estimation. For example, Wu (Wu et al., 2016) compared stepwise linear regression, k-nearest neighbors (KNN), SVR, RF, and stochastic gradient boosting (SGB) methods, and used Landsat imagery to estimate the forest biomass in the northwest region of Zhejiang, China. The results found that the RF method performed the best. Xu (Xu et al., 2018) utilized QuickBird imagery to gather data from the Houbaisha forest region in Fujian Province and established a BP artificial neural network to estimate forest biomass. Zhang (Zhang et al., 2020) conducted an assessment of eight machine learning methodologies, which included multivariate adaptive regression splines (MARS), SVR, RF, categorical boosting (CatBoost), multilayer perceptron (MLP), etc., by estimating forest biomass for performance comparison. The results indicated that the CatBoost algorithm outperformed the other algorithms in terms of performance.

Numerous researches have demonstrated that BPNN is a commonly utilized conventional machine learning methodology (Xu et al., 2018). RF stands out as one of the superior algorithms when it comes to incorporating learning Bagging strategies (Ma et al., 2023). And CatBoost is a high-performance algorithm in the ensemble learning Boosting strategy (Zhai et al., 2023). However, for a certain research area, there will be significant differences between different machine learning algorithms. Therefore, it is necessary to select three types of machine learning algorithms, attempt to construct various machine learning models for the same research area, and carry out forest AGC estimation. Simultaneously, setting appropriate hyperparameters can enable a model to achieve better generalization capability and optimal performance. However, there is currently a lack of uniform and effective hyperparameters that are particularly suitable for estimating the forest AGC of different regions and scales (Dong et al., 2020a). Consequently, for the regional scale, obtaining the best machine learning algorithm and its best effective hyperparameters will improve the accuracy of forest carbon storage estimation.

Lishui City is the ‘ecological green heart’ of Zhejiang Province. The city boasts a wealth of forest resources, with approximately 80% of its area covered by forests. It has built the Baishanzu National Forest Park which is an important distribution area of subtropical typical forests in Zhejiang Province. Therefore, accurately estimating the forest AGC in Lishui City and analyzing its spatiotemporal dynamics is of great significance for evaluating the contribution of forests in Lishui City to the regional ecosystem carbon and serving the national carbon neutrality strategy. Currently, there is an urgent need for a set of machine learning models and corresponding optimized hyperparameters for the AGC estimation of subtropical forest in Lishui City. Based on this requirement, this study aims to obtain a set of machine learning hyperparameters suitable for subtropical forests in Lishui City and invert the spatiotemporal distribution of the forest AGC in Lishui City. The specific objectives include: (1) selecting appropriate remote sensing variables using the Boruta algorithm, (2) tuning hyperparameters and compaing three kinds of machine learning: BPNN, RF and Catboost, (3) selecting the best hyperparameters and machine learning model to invert the forest AGC distributions, (4) analyzing the spatiotemporal dynamics of the forest AGC in Lishui City. This study offers methodological insights for accurately monitoring subtropical forest carbon storage. Additionally, the results will furnish invaluable data support for comprehending the spatiotemporal distribution of forest AGC in Lishui City and augmenting the forest’s carbon sequestration capacity.

## 2 Materials and methods

### 2.1 Study area

**Figure 1:**
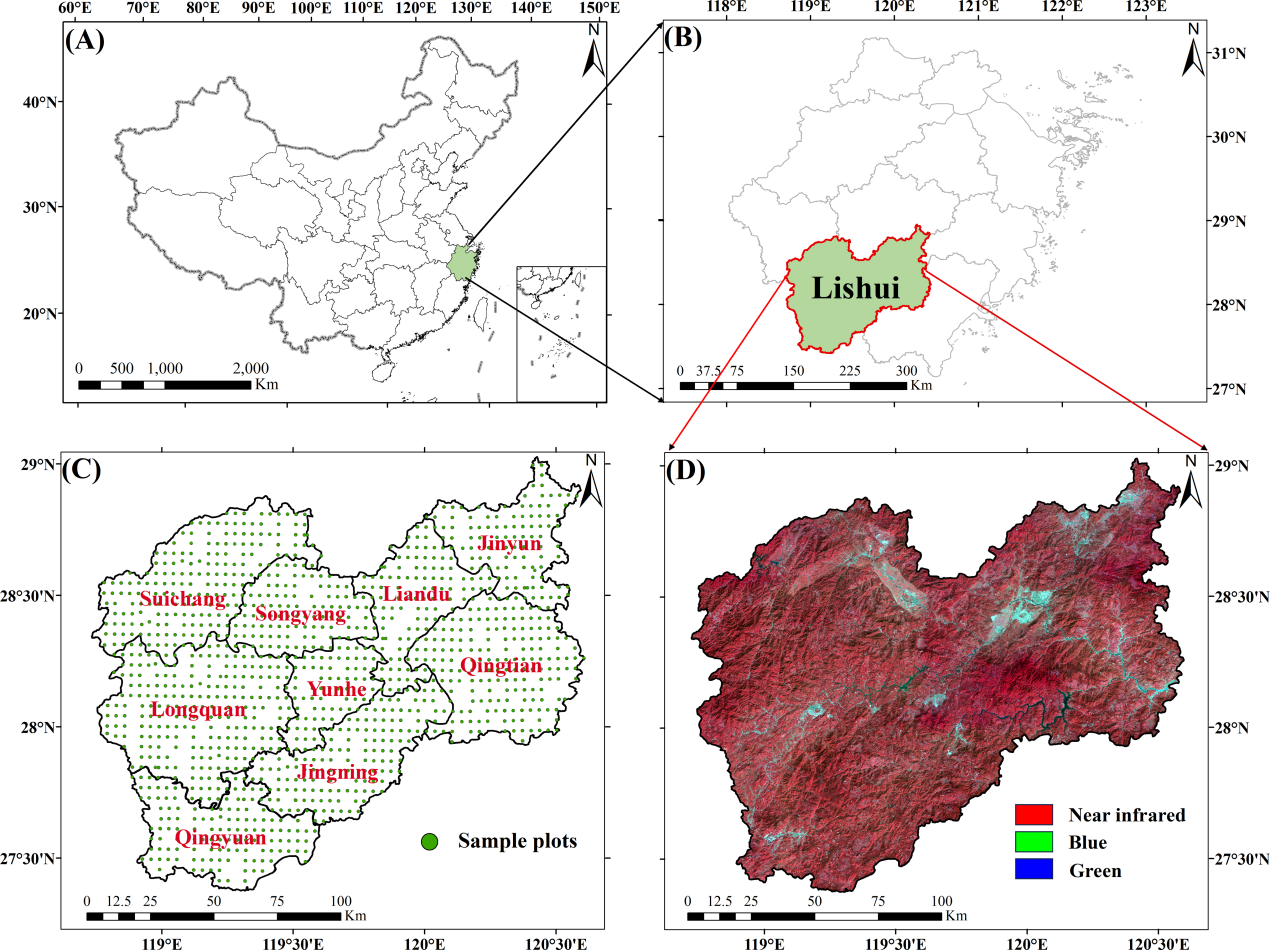
Study area: **(A, B)** Location of Lishui City; **(C)** Spatial distribution of sample plots for continuous forest inventory in Lishui City from 1989 to 2019; **(D)** Landsat remote sensing imagery of Lishui City in 2019.

Lishui City (**Figures 1A, B**) is situated in the southwestern part of Zhejiang Province, China, at the conjunction of Zhejiang and Fujian provinces (27°25′N~28°57′N, 118°41′E~120°26′E). The total area reaches 1.73×10^6^ ha (Diao et al., 2022; Xiong et al., 2021). The terrain of Lishui City is mainly characterized by hills and mountains. The climate in the region exhibits characteristics of a subtropical maritime monsoon. Lishui is the first ecological city in Zhejiang Province, known as the “ forest sea of southern Zhejiang “. The forest area of Lishui accounts for 21.98% of the total forest area in Zhejiang Province. Lishui has a forested area of 5.8×10^5^ ha, accounting for 80.13% of the city area, with a total volume of 103.82 million m^3^ of standing trees and a forest coverage rate of 81.70% (Xiong et al., 2020). Lishui City is abundant in forest resources, housing a diverse array of plant species. The predominant types of forests in the area are coniferous forests, broad-leaved forests, and bamboo forests. According to the Zhejiang Province Forest Land Protection and Utilisation Plan (2017–2020) and the National Forest Plan (2016–2050) (Huang et al., 2020; Li et al., 2018; Zhang et al., 2019), land-use types in Lishui City were classified into six categories, including urban land, water body, cultivated land, broad-leaved forest, needle-leaved forest and bamboo forest. In 2019, the area of coniferous forests accounted for 43.99%, broad-leaved forests accounted for 31.59%, and bamboo forests accounted for 8.90%.

### 2.2 Datasets and processing

#### 2.2.1 Remote sensing data

This study uses Landsat5 TM data from 1989 to 2009 (every 5 years) and Landsat8 OLI data from 2014 and 2019 (**Figure 1D**) to quantitatively estimate the forest carbon storage in Lishui City. GEE possesses an extensive collection of remote sensing data and robust parallel processing abilities (Li and Xu, 2021). The use of GEE greatly saves data processing time and improves data processing accuracy. Therefore, this study extracts the required remote sensing images based on the GEE cloud platform. The GEE platform performs radiometric calibration and atmospheric correction on image data from various sensors to enhance consistency and minimize the impact of different sensors on inversion results (Gorelick et al., 2017; Xie et al., 2020). Additionally, GEE harmonizes the wavebands of these sensors by selecting similar bands for analysis, further reducing sensor-related discrepancies and improving overall data consistency (Wang et al., 2020). The criteria for image selection are cloud cover less than 10% and image acquisition time in summer (June to September). The main Landsat orbit numbers covering Lishui City are 119/040 and 119/041. To mitigate the pseudo-changes in spectral features caused by cloud cover, all clouds and cloud shadows in the images are eliminated using the CFMask algorithm. Clear observations from nearby months are used to fill in the gaps. After processing, a total of 192 Landsat TM and OLI images were collected. These images were then synthesized into 7 periods of 14 scenes of remote sensing images through annual median values and were stitched and cropped according to administrative regions. This resulted in 7 clear, cloud-free Landsat remote-sensing images of Lishui City. These remote sensing images are used for land use remote sensing classification in Lishui City, construction of AGC remote sensing estimation models, and more.

#### 2.2.2 Processing observed data

This study uses the continuous forest resource survey data of Zhejiang Province from seven periods: 1989, 1994, 1999, 2004, 2009, 2014, and 2019 as the measured data. The continuous survey of forest resources in Zhejiang began in 1979, using a systematic sampling method to set up sample plots (**Figure 1C**). Plots are set up at the intersections of a grid with a north-south interval of 4 km and an east-west interval of 6 km. The plot area is 0.08 ha (28.28 m × 28.28 m). In the process of conducting a forest resource assessment, data such as the height of the trees, the diameter at chest level, and the breadth of the tree canopy are documented. By integrating the allometric growth formulas specific to various tree species, the AGC for each tree is computed. Subsequently, the AGC density for every plot is determined (Yang et al., 2022). This study uses the method of three times the standard deviation (Ribal and Young, 2020) to eliminate outliers. A total of 1616 plot data were finally obtained. The number of plots used to build the model each year and the statistics are shown in **Table 1**. The annual plot data is randomly segregated into training and testing subsets in a 7:3 ratio. The training subset is used to train the AGC prediction model, whereas the testing subset is employed to validate the accuracy of the model.

**Table 1:** Statistical information of forest in sample plots in Lishui City.

Year	Number of plots	Minimum (Mg C ha^-1^)	Maximum (Mg C ha^-1^)	Mean (Mg C ha^-1^)	Standard Deviation (Mg C ha^-1^)
1989	262	0.04	43.65	9.11	7.16
1994	228	0.06	38.89	9.23	6.13
1999	180	0.30	34.37	12.49	5.97
2004	265	0.18	47.78	14.01	8.69
2009	253	1.86	58.38	21.82	10.43
2014	241	7.68	73.30	31.69	15.45
2019	194	0.21	69.58	30.31	18.18

## 3 Research methodology

### 3.1 Remote sensing variable settings

The remote sensing parameters gathered in this research encompass distinctive variables like original bands, tassel cap transformation, vegetation index, and texture information, as shown in **Table 2**. The tasseled cap transformation is capable of effectively segregating spectral information associated with vegetation growth and senescence (Hadi et al., 2016; Mostafiz and Chang, 2018). Vegetation indices, which quantify vegetation cover and health status by integrating reflectance from distinct spectral bands, are indicative metrics (Macintyre et al., 2020). These indices demonstrate a strong correlation with vegetation biomass. Analyzing the GLCM can lead to a more precise identification of vegetation structure (Chrysafis et al., 2019), thus assisting in the estimation of forest AGC. These four types of features are key parameters in estimating forest AGC. The original bands are six spectral bands: blue (B, 0.45–0.52 μm), green (G, 0.52–0.60 μm), red (R, 0.63–0.69 μm), near-infrared (NIR, 0.77–0.90 μm), short-wave infrared 1 (SWIR1, 1.55–1.75 μm), and short-wave infrared 2 (SWIR2, 2.08–2.35 μm). The tasseled cap transformations include tasseled cap blue (TCB), tasseled cap green (TCG), tasseled cap wetness (TCW), and tasseled cap angle transformation (TCA). Vegetation indices include the commonly used difference vegetation index (DVI), normalized difference vegetation index (NDVI), enhanced vegetation index (EVI) ratio vegetation index (RVI), enhanced vegetation index (EVI), and ratio vegetation index (RVI), and 11 other indices. The texture information contains nine pieces of information such as variance, contrast, dissimilarity, correlation, etc., in which the texture features are extracted from five windows of size 3×3, 5×5, 7×7, 9×9 and 11×11. Based on the four original waveforms acquired under the five windows, we obtained 180 texture features. In summary, the feature variables acquired were 6 raw bands, 11 vegetation indices, 4 tassel cap transformations, and 180 texture features, totaling 201 remotely sensed variables.

**Table 2:** Remote sensing variables used for estimating forest AGC density.

Feature Types	Feature Names	Descriptions	Notes and References
Original bands	Blue band (B)	/	/
	Green band (G)		
	Red band (R)		
	Near-infrared band (NIR)		
	Shortwave infrared 1 band (SWIR1)		
	Shortwave infrared 2 band (SWIR2)		
Tasseled cap transformation	Angle (TCA)	0.3037 × B + 0.2793 × G + 0.4743 × R + 0.5585 × NIR + 0.5082 × SWIR1 + 0.1863 × SWIR2	(Sharaf El Din, 2020)
	Brightness (TCB)	0.2043×B+0.4158×G+0.5524×R+0.5741×NIR+0.3124×SWIR1+0.2303×SWIR2	
	Greenness (TCG)	−0.1603×B−0.2819×G−0.4934×R+0.7940×NIR−0.0002×SWIR1−0.1446×SWIR2	
	Wetness (TCW)	0.0315×B+0.2021×G+0.3102×R+0.1594×NIR−0.6806×SWIR1−0.6109×SWIR2	
Spectral vegetation indices	Difference vegetation index (DVI)	NIR−R	(Alvarenga et al., 2023)
	Normalized difference vegetation index (NDVI)	$\frac{\text{NIR}-\text{R}}{\text{NIR}+\text{R}}$	(Zhen et al., 2021)
	Ratio Vegetation Index (RVI)	$\frac{NIR}{R}$	(Tan et al., 2019)
	Modified Normalized Difference Water Index (MNDWI)	$\frac{G-SWIR1}{G+SWIR1}$	(Qiu et al., 2022)
	Normalized Difference Built-up Index (NDBI)	$\frac{SWIR1-NIR}{NIR+SWIR1}$	(Muhaimin et al., 2022)
	Normalized Difference Moisture Index (NDMI)	$\frac{NIR-SWIR1}{NIR+SWIR1}$	(Li et al., 2017)
	Normalized Difference Infrared Index (NDII)	$\frac{SWIR2-NIR}{NIR+SWIR2}$	(Sriwongsitanon et al., 2016)
	Chlorophyll Vegetation Index (CVI)	$\frac{NIR*R}{G*G}$	(Maudhi et al., 2023)
	Transformed Vegetation Index (TVI)	60×(NIR−R)−100×(R−G)	(Xing et al., 2019)
	Soil Adjusted Vegetation Index (SAVI)	$\frac{1.5\times \left(NIR-R\right)}{NIR+R+0.5}$	(Zhen et al., 2021)
	Enhanced vegetation index (EVI)	$2.5\times \frac{\text{NIR}-\text{R}}{\text{NIR}+6\times \text{R}-7.5\times \text{B}+1}$	(Zhen et al., 2021)
Texture features based on the gray-level co-occurrence matrix (GLCM)	Angular second moment (ASM)	$\sum_{i=1}^{n}\sum_{j=1}^{n}P{\left(i,j\right)}^{2}$	$P\left(i,j\right)=\frac{V\left(i,j\right)}{\sum_{i=1}^{n}\sum_{j=1}^{n}V\left(i,j\right)}$ V(i,j) is the i th row of the j th column in the N th moving window. $u_{x}=\sum_{j=1}^{n}j\sum_{i=1}^{n}P\left(i,j\right)$ $u_{y}=\sum_{i=1}^{n}i\sum_{j=1}^{n}P\left(i,j\right)$ $\sigma_{x}=\sum_{j=1}^{n}{\left(j-u_{i}\right)}^{2}\sum_{i=1}^{n}P\left(i,j\right)$ $\sigma_{y}=\sum_{i=1}^{n}{\left(i-u_{j}\right)}^{2}\sum_{j=1}^{n}P\left(i,j\right)$ (Bharati et al., 2004; Dong et al., 2020b; Huang et al., 2023; Iqbal et al., 2021; Mohammadpour et al., 2022)
	Contrast (CON)	$\sum_{\left|i-j\right|=0}^{n-1}{\left|i-j\right|}^{2}\left\{\sum_{i=1}^{n}\sum_{j=1}^{n}P\left(i,j\right)\right\}$	
	Inverse Difference Moment (IDM)	$\sum_{i,j=0}^{N-1}\frac{{\text{P}}_{\text{i},\text{j}}}{1+{\left(\text{i}-\text{j}\right)}^{2}}$	
	Correction (COR)	$\sum_{i,j=0}^{N-1}i\frac{\sum_{i,j}^{N-1}ijP_{i,j}-\mu_{i}\mu_{j}}{\sigma_{i}^{2}\sigma_{j}^{2}}$	
	Variance (VAR)	$\sum_{i=1}^{n}\sum_{j=1}^{n}{\left(i-\mu \right)}^{2}P\left(i,j\right)$	
	Entropy (ENT)	$-\sum_{i=1}^{n}\sum_{j=1}^{n}P\left(i,j\right)\log\left(P\left(i,j\right)\right)$	
	Dissimilarity (DIS)	$\sum_{\left|i-j\right|=0}^{n}\left|i-j\right|\left\{\sum_{i=1}^{n}\sum_{j=1}^{n}P\left(i,j\right)\right\}$	
	Cluster Shade (SHA)	$\sum_{i=1}^{n}\sum_{j=1}^{n}{\left(i+j-\mu_{i}-\mu_{j}\right)}^{3}P\left(i,j\right)$	
	Cluster prominence (PRO)	$\sum_{i=1}^{n}\sum_{j=1}^{n}{\left(i+j-\mu_{i}-\mu_{j}\right)}^{4}P\left(i,j\right)$	

### 3.2 Feature variable selection

Feature selection refers to the procedure of choosing the most impactful attributes from a collection of attributes to diminish the dimensionality of the attribute space (Wan et al., 2020). The main purpose of feature selection is to remove redundant or prediction-irrelevant features (Jamei et al., 2023). The Boruta feature variable selection method obtains shadow features by reordering the original features (Liu et al., 2021), and ensures that the quantity of shadow attributes remains equivalent to the original attributes. The RF model is subsequently employed to train both the original and shadow attributes and to compute the significance score for each attribute. After multiple iterations, the Boruta algorithm compares the importance scores of the original features and shadow features to achieve importance evaluation (Habibi et al., 2023; Prasad et al., 2019). If the significance score of the original attribute surpasses that of the shadow attribute, the attribute is deemed significant. Conversely, if not, the attribute is deemed unimportant. All original features are marked as important or not important. The output is the dataset of all important features calculated. This dataset is the result of variable selection. This study uses the Boruta algorithm to reduce the dimensionality of 201 remote sensing variables for each year. The features marked as important by Boruta are selected as the variables for building different machine-learning algorithms each year.

### 3.3 AGC model construction scheme and method

Following the outcomes of variable selection, three machine learning methodologies, namely BPNN, RF and CatBoost, are employed to formulate the forest AGC prediction model and fine-tune the parameters. The precision of the three models is assessed and juxtaposed in light of the annual results. Ultimately, a model that exhibits superior performance and robust generalization capabilities is chosen. It is used as the final model for estimating and inverting the spatiotemporal distribution of the forest AGC in Lishui City.

#### 3.3.1 BPNN algorithm

BPNN is a multi-layer feedforward neural network. It boasts benefits such as elevated precision, potent generalization capabilities, commendable adaptability, and minimal computational intricacy. It is highly applicable for estimating forest AGC. Its fundamental concept is gradient descent, which employs gradient search techniques to minimize the mean squared discrepancy between the network’s actual output value and the anticipated output value (Eshraghian et al., 2023). First, the network parameters are initialized, and the network model is constructed. Subsequently, the training sample collection is fed into the network. By calculating the loss function, the optimization of each node and weight of the neural network is carried out. If the mean squared discrepancy between the real output value and the anticipated output value is excessively large, the weights are adjusted sequentially from the output layer back to the input layer utilizing the backpropagation algorithm. By perpetually fine-tuning the connection weights among layers and the node thresholds, the network’s output is brought nearer to the projected output (Huang et al., 2019). This study uses the Python Keras library to build the BPNN model, with the main hyperparameters including alpha and max_iter.

#### 3.3.2 RF algorithm

The RF algorithm is a non-parametric combinatorial algorithm based on classification and regression decision trees (Su et al., 2020). It can handle the complex non-linear relationship between forest AGC and remote sensing variables (Samadianfard et al., 2022). At the same time, it has a low sensitivity to noise present in the training samples, can effectively deal with the accuracy reduction problem caused by data missing, and can also identify the importance of prediction variables (Cai et al., 2020). This model builds a series of base learners through resampling, and finally outputs the prediction results of these base learners through voting, thus taking into account the ability to solve regression and classification problems (Elbeltagi et al., 2023; Li X, et al., 2023; Zhang C, et al., 2023). This study uses the Python Scikit-learn library to build the RF model to estimate AGC, with the main hyperparameters considered including n_estimators, max_depth, min_samples_split, min_samples_leaf.

#### 3.3.3 CatBoost algorithm

CatBoost is a gradient-boosting algorithm library and is one of the mainstream models of gradient-boosting regression trees (GBRT) (Joo et al., 2023). The advantage of the CatBoost algorithm is that it overcomes gradient bias and effectively solves the problem of prediction offset, improves the accuracy of the algorithm, enhances generalization ability, and can prevent the occurrence of overfitting (Zhang Y, et al., 2023). It employs algorithm amalgamation with symmetric decision trees as foundational learners. It utilizes identical features for bifurcation at every layer during the operational process and computes the leaf node value by minimizing the sample loss on the leaf nodes (Zhong et al., 2023). This study uses the Python catboost to build the CatBoost model, with the key hyperparameters including depth, learning_rate, and l2_leaf_reg.

#### 3.3.4 Parameter optimization of machine learning algorithms

The parameters of an algorithm can influence the effectiveness of the constructed model, so optimizing the parameters of each algorithm is crucial. For different models, this study optimized their key hyperparameters. The key hyperparameters and optimization configurations of each algorithm are shown in **Table 3**. The original data is partitioned into a training dataset (70%) and a testing dataset (30%). The GridSearchCV function, in conjunction with 5-fold cross-validation (Hajihosseinlou et al., 2023), is employed for the optimization of hyperparameters. The ideal combination of hyperparameters for the AGC modeling algorithm is ascertained based on the scoring criterion of the smallest value of the root mean square error (RMSE). The training dataset is utilized to educate the AGC model, while the testing dataset serves to validate its AGC prediction performance. To guarantee that the outcomes of various AGC modeling algorithms are not influenced by the partitioning of the training and testing datasets, the procedure of AGC model hyperparameter adjustment and model performance appraisal is reiterated 1000 times for training (Zhang et al., 2020).

**Table 3:** Hyperparameters tuned and their configurations for each algorithm.

Algorithm	Library	Hyperparameters Tuned	Meaning	Hyperparameters Configurations
BPNN	Keras	alpha max_iter	The parameter of learning rate. Number of training epochs.	(0.0001, 0.0005, 0.001, 0.005, 0.01) (100, 200, 300, 400, 500)
RF	Scikit-learn	n_estimators max_depth min_samples_split min_samples_leaf	The number of trees in the forest. The maximum depth of the tree. The minimum number of samples required to partition an internal node. The minimum number of samples required for leaf nodes.	(100, 200, 300, 400, 500, 600, 700, 800, 900) (1, 5, 10, 15) (2, 4, 6, 8, 10) (1, 2, 3, 4, 5)
CatBoost	catboost	depth learning_rate l2_leaf_reg	Depth of a tree. Boosting learning rate The L2 regularization parameter	(1, 2, 3, 4, 5, 6, 7, 8, 9, 10) (0.005, 0.01, 0.05, 0.1) (100, 200, 300, 400, 500, 600, 700, 800, 900)

### 3.4 Model accuracy evaluation method

In this study, the accuracy of the model was evaluated using three metrics: coefficient of determination (R^2^) and RMSE (Aslami et al., 2019). Typically, elevated values of R^2^ coupled with diminished values of RMSE signify superior model performance.


$$R^{2}=\frac{\sum_{i=1}^{n}{\left({\hat{y}}_{i}-{\bar{y}}_{i}\right)}^{2}}{\sum_{i=1}^{n}{\left(y_{i}-{\bar{y}}_{i}\right)}^{2}}$$

$$RMSE=\sqrt{\frac{\sum_{i=1}^{n}{\left(y_{i}-{\hat{y}}_{i}\right)}^{2}}{n}}$$

In this context, $y_{i}$ , ${\bar{y}}_{i}$ and ${\hat{y}}_{i}$ represent the observed AGC, the average AGC, and the AGC predicted by the model, respectively, with n being the total number of samples. During the evaluation, an R^2^ value closer to 1 indicates a better fit of the model; a smaller RMSE value signifies a lesser degree of scatter between the actual value and the value predicted by the model (Li H, et al., 2023).

### 3.5 Technical route

**Figure 2:**
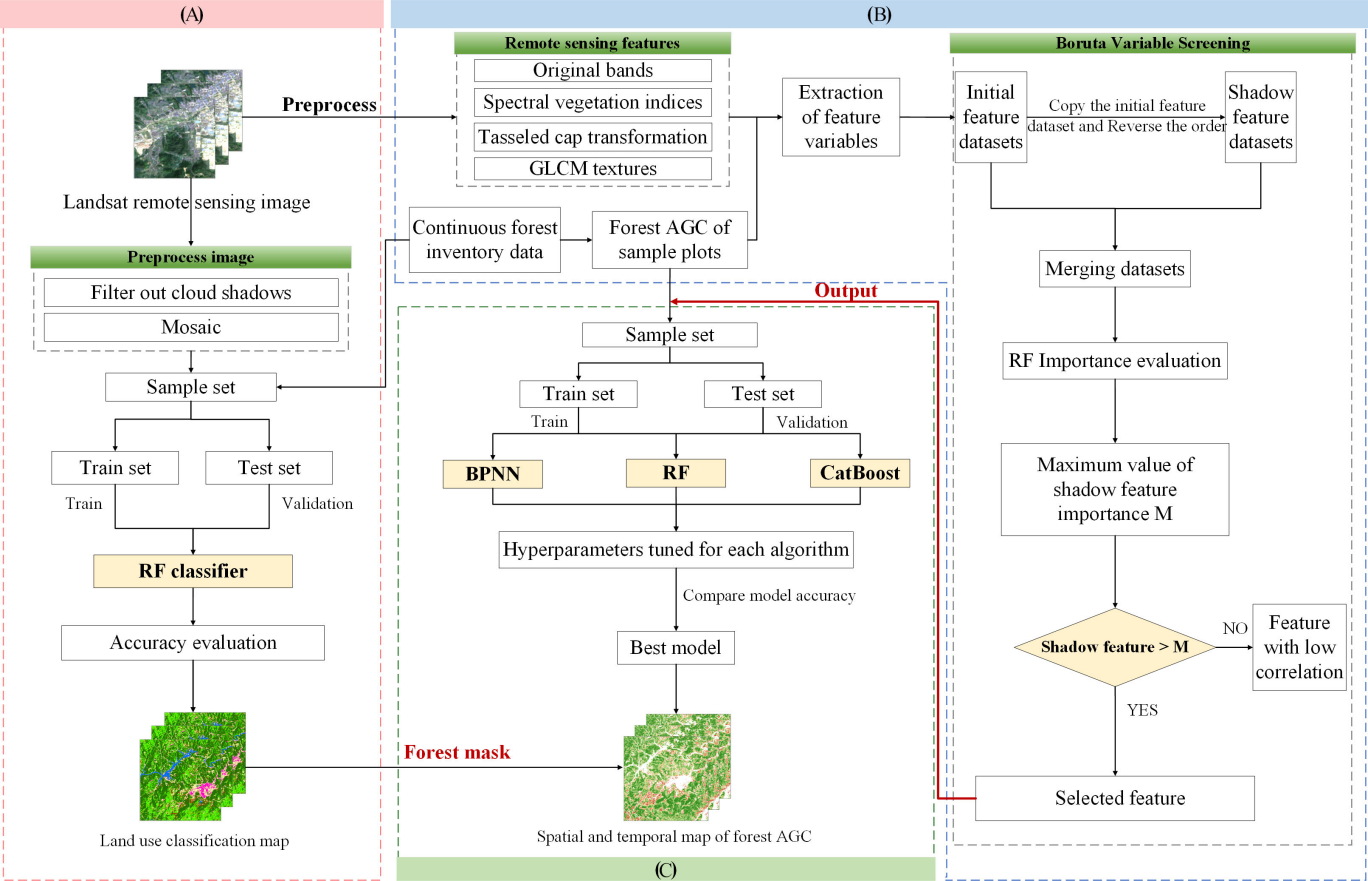
Research technology roadmap: **(A)** land use classification map, **(B)** extraction of remote sensing image information for feature variable selection, **(C)** analyzing the three models, the optimal one was used for the inversion of forest AGC, resulting in a map depicting the spatial and temporal distribution of AGC.

The workflow diagram for estimating the spatial-temporal distribution of forest AGC by integrating land use classification and machine learning algorithms in this study is illustrated in **Figure 2**, primarily comprising three components: (1) Based on the Landsat images from 1989–2019, the RF classifier is used to obtain the land use classification map of Lishui City from 1989–2019. (2) Combining the Landsat image feature variables from 1989–2019 and the continuous forest resource survey data of Zhejiang Province, the Boruta algorithm is used for remote sensing variable selection. This results in variables that are highly related to forest AGC each year. (3) The filtered remote sensing parameters are fed into the BPNN, RF, and CatBoost machine learning algorithms for AGC estimation. The accuracy of the three models is compared, and the optimal model is selected for regional inversion. The corresponding land use classification map is used for masking each year. The final result is the spatio-temporal distribution map of forest AGC in Lishui City.

## 4 Results and analyses

### 4.1 Information on forest distribution in Lishui City

**Figure 3:**
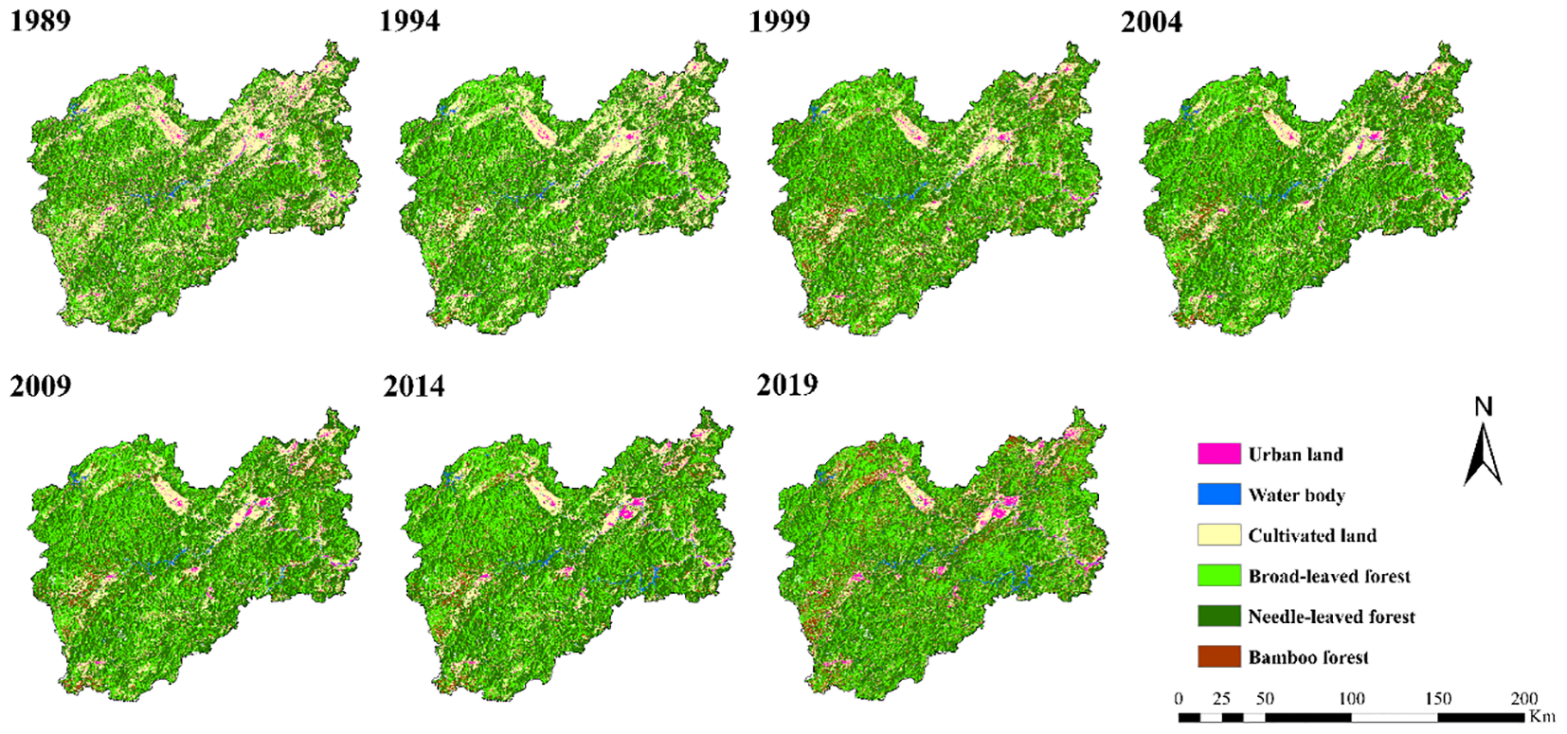
Land use classification map of Lishui from 1989 to 2019.

Based on the GEE platform, the RF classifier is used to classify the land use in Lishui City, the categorization outcomes from seven intervals are displayed in **Figure 3**. Lishui City has rich forest resources, and the proportion of forest area is large. Broad-leaved forests are predominantly found in the southern, western, and central-eastern areas of Lishui City. The western region primarily hosts coniferous forests. Bamboo forests are typically found in proximity to coniferous forests and agricultural areas. Comparing the seven-period classification map (**Figure 3**), it can be seen that from 1989 to 2019, the area of broad-leaved forests in the northwestern and eastern regions increased significantly, while the area of coniferous forests and cultivated land decreased. The area of cultivated land in the northeastern region decreased significantly, urban land increased, and the expanses of broad-leaved forests, coniferous forests, and bamboo forests have seen growth. The area of bamboo forests and broad-leaved forests in the southwestern region increased, while the area of cultivated land and coniferous forests decreased.

The classification results are evaluated for accuracy through forest resource inventory data and manual visual interpretation (**Table 4**). The overall accuracy (OA) of the seven stages is all above 85%. The classification of broad-leaved forests, coniferous forests, and bamboo forests all have a user accuracy (UA) and producer accuracy (PA) exceeding 70%. This indicates that the classification results of each year are relatively accurate. The spatiotemporal distribution information of broad-leaved forests, coniferous forests, and bamboo forests is extracted from it. They are combined into the forest distribution information of seven periods. It is used for the extraction of the forest AGC spatial distribution mask in the later stage.

**Table 4:** Evaluation of land use accuracy over 7 periods.

Year		UL	WB	CL	BLF	NLF	BF	OA	KC
1989	UA	99.63%	90.00%	85.10%	88.50%	94.26%	88.97%	89.81%	0.8641
	PA	86.82%	100.00%	82.09%	83.78%	94.58%	82.20%		
1994	UA	98.15%	90.97%	72.86%	93.66%	97.38%	88.43%	90.48%	0.7912
	PA	91.72%	100.00%	89.25%	74.30%	95.93%	86.99%		
1999	UA	82.29%	79.94%	92.31%	93.50%	97.14%	83.45%	90.91%	0.8797
	PA	93.21%	100.00%	75.32%	82.34%	97.05%	96.80%		
2004	UA	79.33%	85.07%	69.58%	98.90%	95.01%	87.21%	86.61%	0.8325
	PA	67.92%	95.70%	73.57%	77.92%	97.38%	82.87%		
2009	UA	80.88%	85.18%	72.49%	97.20%	94.84%	82.47%	86.68%	0.8338
	PA	93.48%	100.00%	75.56%	70.10%	96.05%	81.94%		
2014	UA	78.92%	86.52%	71.86%	99.72%	94.71%	87.65%	87.08%	0.7776
	PA	94.71%	100.00%	74.07%	70.08%	98.56%	81.87%		
2019	UA	80.88%	86.52%	87.27%	99.69%	83.88%	88.46%	86.74%	0.8367
	PA	93.22%	98.77%	68.57%	71.97%	98.67%	86.61%		

UL, urban land; WB, water body; CL, cultivated land; BLF, broad-leaved forest; NLF, needle-leaved forest; BF, bamboo forest; UA, user’s accuracy; PA, producer’s accuracy; OA, overall accuracy; KC, kappa coefficient.

### 4.2 Variable selection results

Based on the Boruta algorithm, the best combination of remote sensing feature variables selected for each year is shown in **Table 5**. Among them, 21 variables were selected in 1989, 26 variables in 1994, 41 variables in 2004, 47 variables in 2009, 37 variables in 2014, and 45 variables in 2019.

**Table 5:** Characteristic variables were selected based on the Boruta method.

Year	Number of Selected Variables	Name of Selected Variables
1989	21	CVI, NDBI, NDII, NDMI, B1, B1CON, B1DIS, B1PRO, B1VAR, B2, B3, W1B3PRO, W2B3PRO, W1B3VAR, W2B3VAR, W3B3VAR, W1B4SHA, B5, TCA, TCB, TCW
1994	26	NDVI, B1, W3B1DIS, W2B1VAR, B2, W1B2CON, W3B2CON, W3B2DIS, B2IDM, W2B2PRO, W3B2PRO, W4B2PRO, W3B2VAR, W4B2VAR, B3, W2B3CON, W3B3CON, W4B3CON, W2B3DIS, W2B3PRO, W3B3PRO, W4B3PRO, W3B3VAR, W4B3VAR, W4B4CON, TCA
1999	30	CVI, MNDWI, NDBI, NDII, NDMI, NDVI, RVI, B1, W4B1SHA, B2, W3B2IDM, W4B2IDM, B3, W1B3ASM, W2B3ASM, W4B3ASM, W1B3ENT, W2B3ENT, W4B3ENT, W3B3IDM, W4B3IDM, B4, W4B4IDM, W4B4SHA, B5, B7, TCA, TCB, TCW, TVI
2004	41	CVI, MNDWI, NDBI, NDII, NDMI, NDVI, RVI, B1, W2B1DIS, W3B1DIS, W4B1DIS, W2B1PRO, W3B1VAR, W4B1VAR, B2, B2CON, W1B2CON, B2DIS, W1B2DIS, W2B2DIS, W3B2DIS, W4B2DIS, W4B2PRO, W4B2VAR, B3, W3B3CON, W4B3CON, W1B3DIS, W2B3DIS, W3B3DIS, W4B3DIS, W3B3PRO, W1B3VAR, W2B3VAR, W3B3VAR, W4B3VAR, B5, B7, TCA, TCB, TCW
2009	47	CVI, DVI, EVI, NDBI, NDII, NDMI, NDVI, RVI, SAVI, B1, W4B1ASM, W4B1CON, W3B1DIS, W4B1DIS, W4B1ENT, W4B1IDM, W1B1PRO, W2B1PRO, W1B1VAR, W2B1VAR, B2, W3B2CON, W3B2DIS, B3, W4B3ASM, W2B3CON, W3B3CON, W4B3CON, W1B3DIS, W2B3DIS, W3B3DIS, W4B3DIS, W4B3ENT, W3B3IDM, W4B3IDM, W1B3PRO, W4B3PRO, W1B3VAR, W3B3VAR, W4B3VAR, B5, B7, TCA, TCB, TCG, TCW, TVI
2014	37	CVI, EVI, NDVI, RVI, B2, W2B2CON, W3B2CON, W4B2CON, W2B2DIS, W3B2DIS, W4B2DIS, W4B2IDM, W2B2PRO, W3B2PRO, W4B2PRO, W3B2VAR, W4B2VAR, B3, W4B3DIS, B4, W2B4CON, W3B4CON, W4B4CON, W2B4DIS, W3B4DIS, W4B4DIS, W4B4IDM, W2B4PRO, W3B4PRO, W4B4PRO, W4B4SHA, W2B4VAR, W3B4VAR, W4B4VAR, TCA, TCG, TVI
2019	45	CVI, NDBI, NDII, NDMI, B2, W2B2CON, W3B2CON, W4B2CON, W2B2DIS, W4B2DIS, W3B2IDM, W4B2IDM, W2B2PRO, W3B2PRO, W2B2SHA, W3B2VAR, W4B2VAR, B3, W2B3DIS, W4B3IDM, W4B3VAR, B4, W2B4CON, W3B4CON, W4B4CON, W2B4DIS, W3B4DIS, W4B4DIS, W2B4PRO, W3B4PRO, W4B4PRO, W2B4VAR, W3B4VAR, W4B4VAR, W2B5CON, W3B5CON, W4B5CON, W2B5DIS, W3B5DIS, W4B5DIS, W3B5VAR, B6, B7, TCA, TCW

WiBjxx is the texture information for the jth band window size i of the image; xx refers to MEA, VAR, SHA, CON, IDM, DIS, ENT, ASM, and PRO.

### 4.3 AGC model construction and prediction results

According to the three machine learning hyperparameter configurations shown in **Table 3**, parameter optimization was performed on the seven-period data (5 years per period) of the three machine learning models respectively. The optimal hyperparameter combination for each AGC estimation model was determined through a cross-validation grid search. Using the minimum root mean square value as the scoring criterion, a total of 1000 trainings were conducted (**Table 6**). After determining the best hyperparameter combination, the forest AGC estimation model was constructed.

**Table 6:** Optimal values for each hyperparameter for each year.

Year		1989	1994	1999	2004	2009	2014	2019
BPNN	alpha	0.01	0.0001	0.0001	0.01	0.01	0.0001	0.005
	max_iter	300	200	300	200	100	200	300
	learning_rate	0.1	0.1	0.1	0.1	0.1	0.1	0.1
RF	n_estimators	200	400	300	300	700	200	300
	max_depth	10	10	15	10	10	10	15
	min_samples_split	2	2	2	2	2	4	4
	min_samples_leaf	2	2	2	2	1	1	1
CatBoost	depth	1	2	2	1	2	2	2
	learning_rate	0.1	0.1	0.1	0.1	0.1	0.1	0.1
	l2_leaf_reg	100	100	100	200	200	200	100

**Figure 4:**
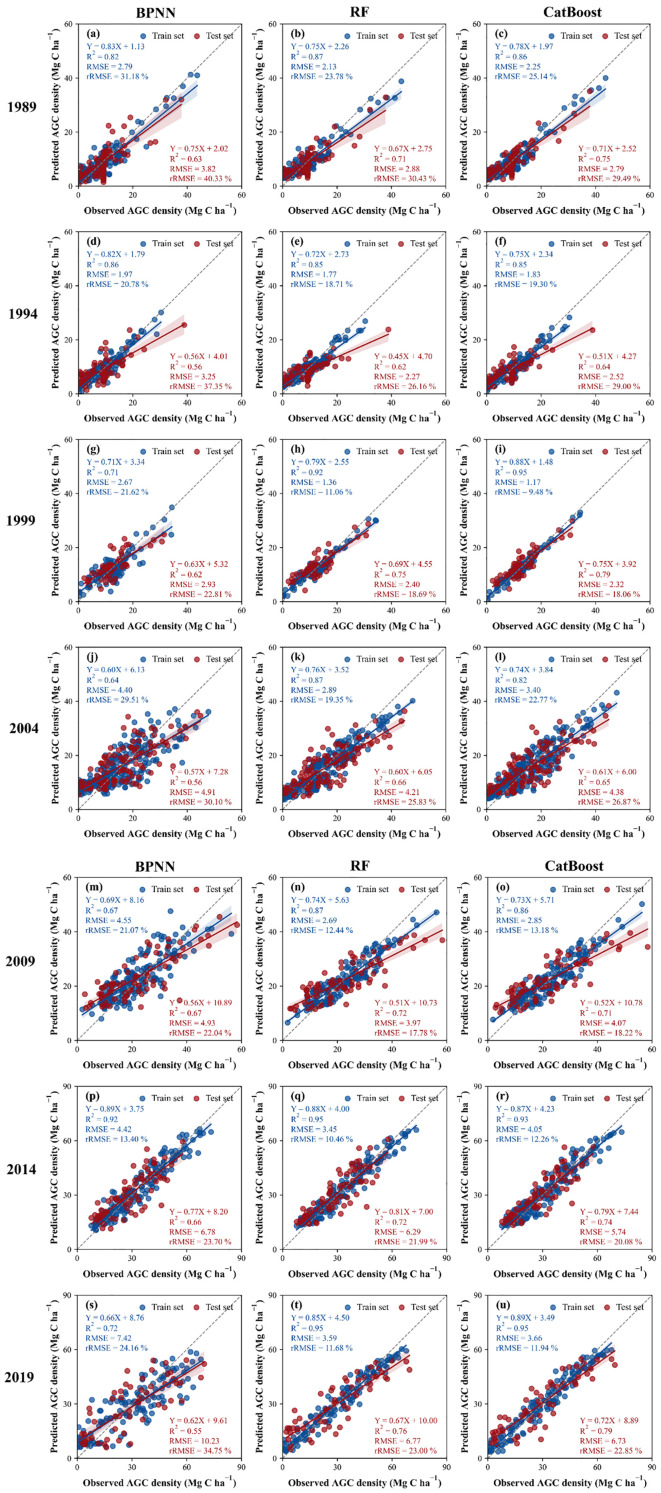
**(A–U)** Accuracy assessment of three Landsat-based AGC prediction models per year.

This study employs three machine learning algorithms, namely BPNN, RF, and CatBoost, to develop three predictive models for forest AGC spanning the years 1989–2019. The correlation between the AGC densities of each year estimated by the three models and the measured AGC densities of the sample plots is shown in **Figure 4**. During 1989–2019, the BPNN model training accuracy R^2^ ranged from 0.64–0.92 with a training RMSE of 1.97–7.42 Mg C ha^-1^, and the testing accuracy R^2^ ranged from 0.55–0.67 with a testing RMSE of 2.93–10.23 Mg C ha^-1^. The RF model training accuracy R^2^ was between 0.85–0.95 and the training RMSE was 1.36–3.59 Mg C ha^-1^, and the testing accuracy R^2^ was between 0.62–0.76 and the RMSE was 2.27–6.77 Mg C ha^-1^. The CatBoost model had a training accuracy R^2^ of between 0.82–0.95 a training phase RMSE of 1.17–4.05 Mg C ha^-1^, and a testing accuracy R^2^ of between 0.64–0.79, and a testing phase RMSE of 2.32–6.73 Mg C ha^-1^. Meanwhile, observing the extent to which the three model fit curves deviate from the 1:1 line reveals that overestimation and underestimation are not prominent when the AGC is in the 0–40 Mg C ha^-1^ range; underestimation generally occurs when the AGC is greater than 40 Mg C ha^-1^. In addition, from 1989 onwards, the AGC density in the sample site showed an increasing trend from year to year.

**Figure 5:**
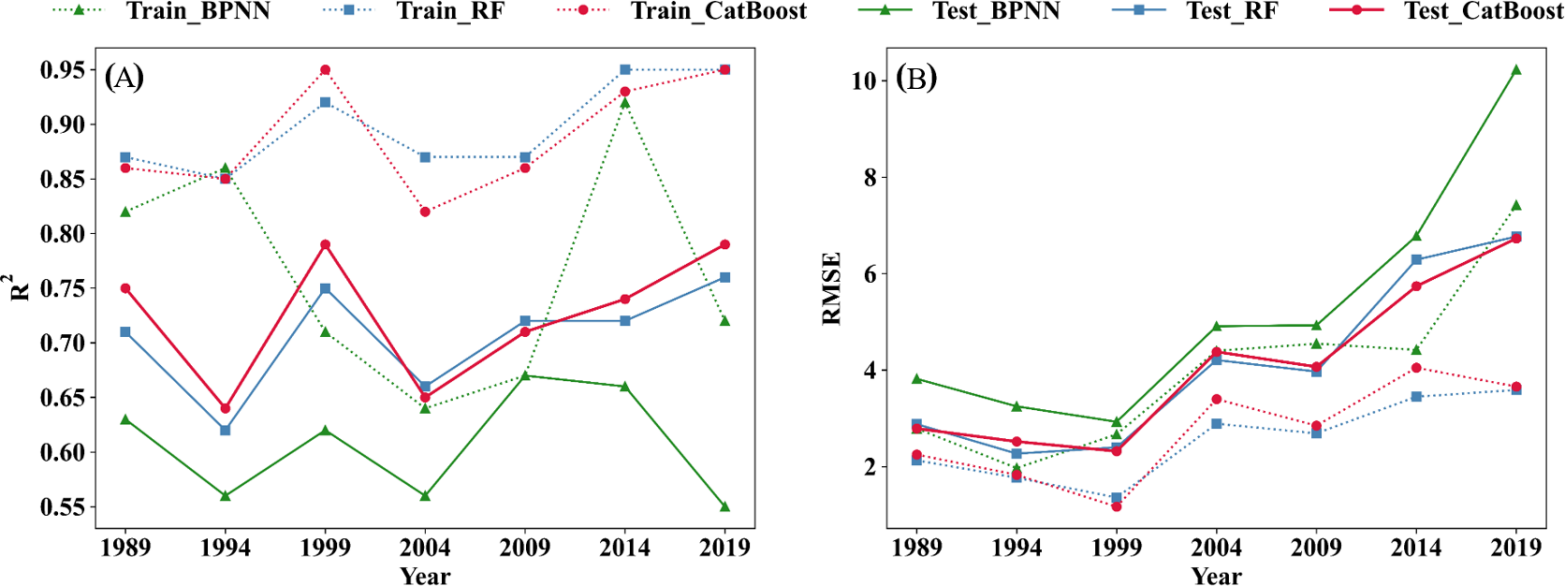
Comparison of the accuracy of the three models. **(A)** the values of the R2 for three models. **(B)** the values of the RMSE for three models.

**Figure 5** shows the accuracy (R^2^) and error (RMSE) of the three models in the training set and test set for each year. The comparison revealed that the CatBoost model had the highest average accuracy R^2^ (R^2^ = mean ± standard deviation) in the test set of all years. The second highest is the RF model (R^2^ = mean ± standard deviation). The average accuracy R^2^ (R^2^ = mean ± standard deviation) of the BPNN model’s test set is the lowest. At the same time, the average RMSE (RMSE=mean ± standard deviation) of the BPNN model’s test set is the highest. The average RMSE of the CatBoost model (RMSE=mean ± standard deviation) and the RF model (RMSE=mean ± standard deviation) is much lower than the average RMSE of BPNN. Therefore, the estimates of CatBoost and RF are superior to BPNN in all years, and the estimates of the CatBoost model are slightly superior to the RF model.

**Figure 6:**
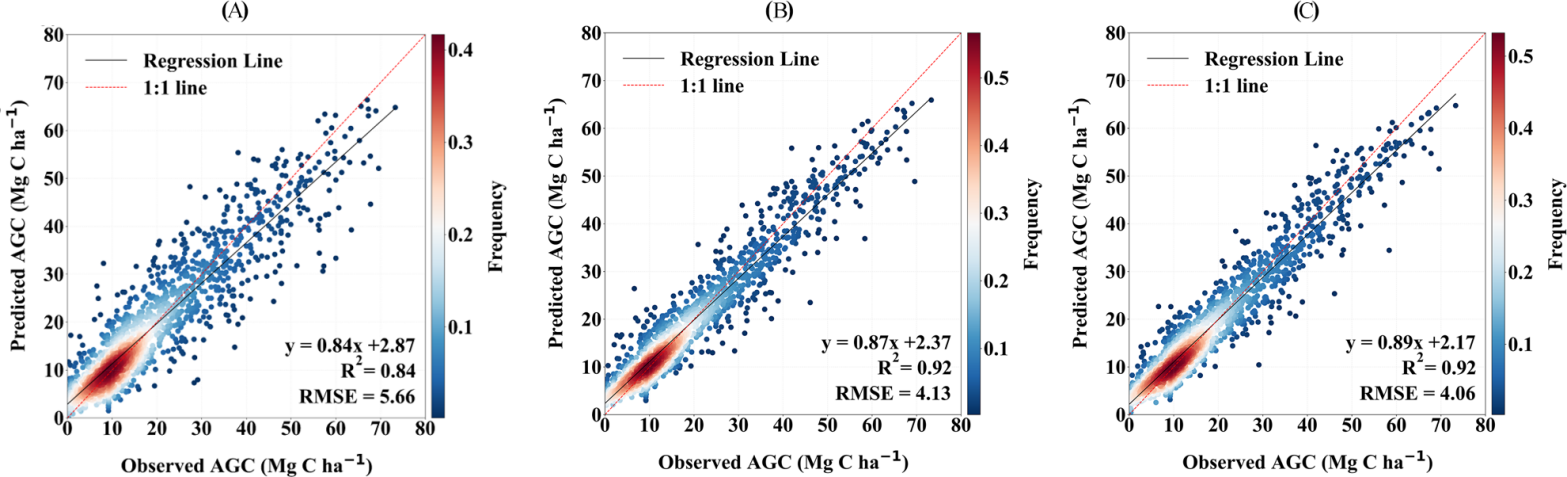
Evaluating the precision of AGC prediction models derived from Landsat: **(A)** BPNN machine learning model, **(B)** RF machine learning model, **(C)** CatBoost machine learning model.

The training and testing results of all sample plots over the years are evaluated, resulting in a scatter plot (**Figure 6**). It can be seen that the training R^2^ of the RF model is the same as the training R^2^ of the CatBoost model, which is 9.2% higher than BPNN. The test R^2^ of the CatBoost model is 2.5% higher than the test R^2^ of the RF model, and 12.16% higher than the test R^2^ of the BPNN model. Among the three models, the RMSE of the CatBoost model in the testing phase is the lowest. It is 3.65% lower than the RF model and 25.96% lower than the BPNN model. The RMSE of the BPNN model in the testing phase is the lowest, 3.65% lower than the RF model, and 25.96% lower than the BPNN model. The range of overestimation and underestimation of the BPNN model is greater than that of the RF model and the CatBoost model.

In summary, the forest AGC predictive model built using the CatBoost algorithm demonstrates superior accuracy and overall performance. Therefore, this study will use the CatBoost model as the best estimation model for the spatiotemporal distribution of the forest AGC in Lishui City.

### 4.4 Spatiotemporal distribution of AGC in Lishui City

**Figure 7:**
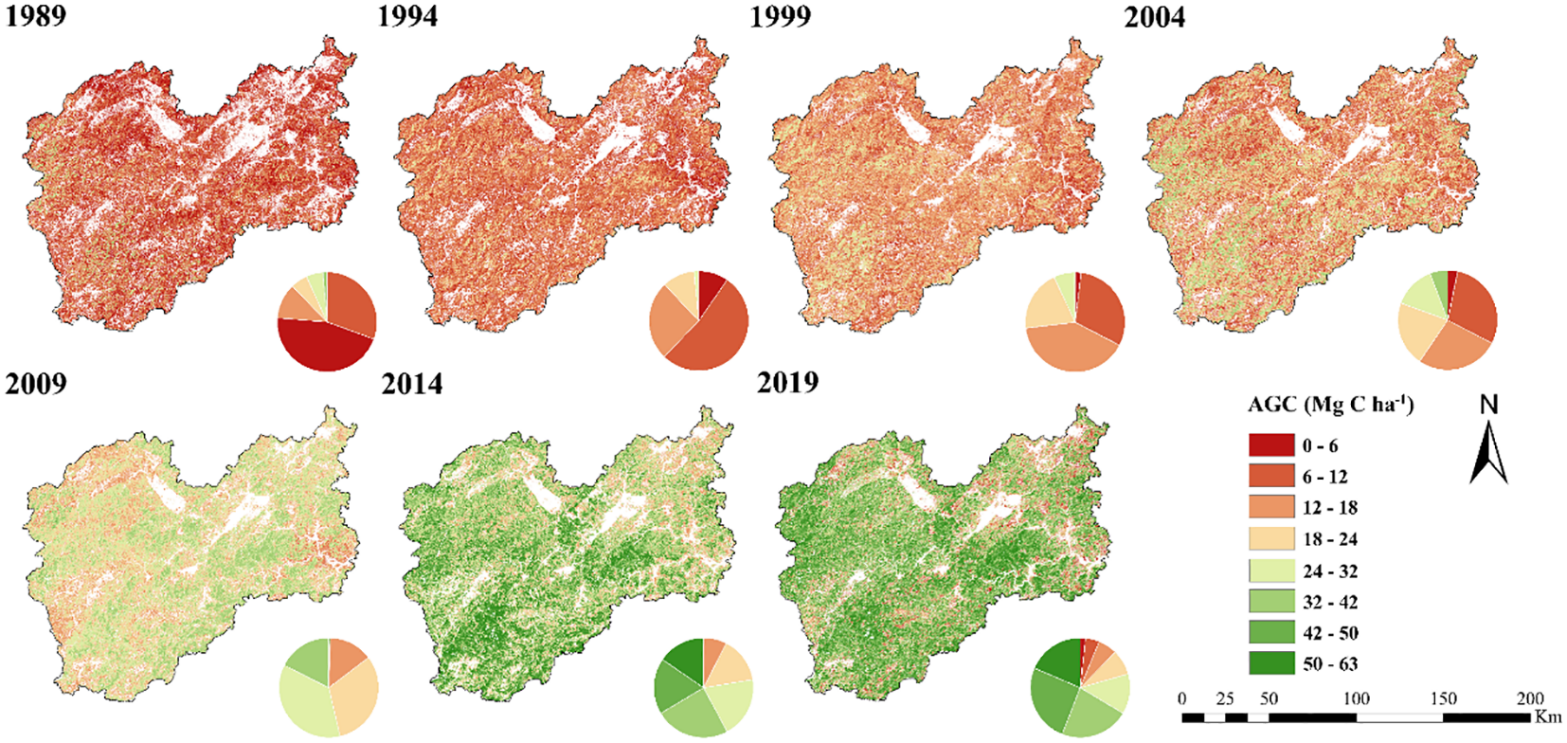
Spatial distribution of AGC on forested land in Lishui from 1989 to 2019.

The dispersion of forest AGC density in Lishui City over the period from 1989 to 2019, estimated based on the CatBoost model, is shown in **Figure 7** From a spatial perspective, the AGC values in the central and southwestern regions of Lishui City are relatively high. Areas with high forest AGC values are distributed in the southwestern part of Suichang County, the northwestern and southeastern parts of Longquan County, the northern part of Qingyuan County, the southeastern part of Songyang County, the northern and southeastern parts of Yunhe County, the northeastern part of Jingning County, the northern and southeastern parts of Jingde County, the southeastern part of Jinyun County, the southwestern part of Qingtian County, and the southern part of Liandu District. These areas have more mountains and a wide forest coverage area, so the forest AGC values are higher. The forest AGC values in areas near the city are generally lower, and the carbon density is also lower. From a temporal perspective, the forest AGC density in Lishui City shows an upward trend. It has changed from being dominated by low AGC values in 1989 to being dominated by high AGC values in 2019. During the period from 1989 to 2004, the proportion of Lishui forests with AGC values of 0–24 Mg C ha^-1^ was higher. The highest percentage of 0–6 Mg C ha^-1^ was 45.71% in 1989, 6–12 Mg C ha^-1^ was 52.63% in 1994, 12–18 Mg C ha^-1^ was 40.44% in 1999 and 6–12 Mg C ha^-1^ was 29.41% in 2004. From 2009 to 2019, Lishui City saw a higher proportion of forest AGC values ranging from 24–63 Mg C ha^-1^. In 2009, the 24–32 Mg C ha^-1^ range had a peak percentage of 36.08%. In 2014, the highest percentage of 24.26% was in the 32–42 Mg C ha^-1^ range. By 2019, the 42–50 Mg C ha^-1^ range had the highest percentage of 25.56%.

**Figure 8:**
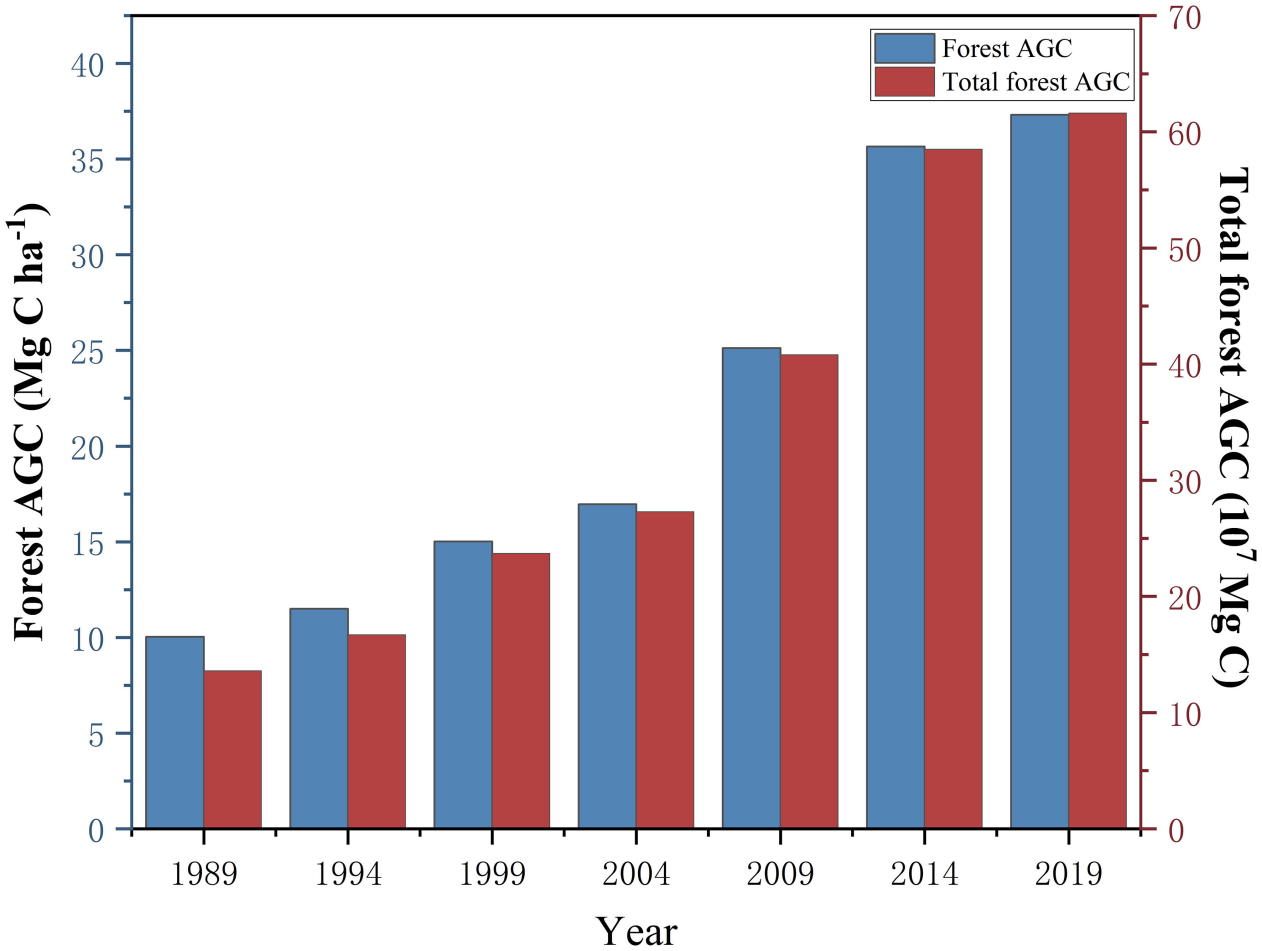
Forest AGC and total forest AGC storage from 1989 to 2019.

As shown in **Figure 8**, the average forest AGC in Lishui City increased from 10.03 Mg C ha^-1^ in 1989 to 37.32 Mg C ha^-1^ in 2019, an increase of 2.72 times. Among them, the growth rate was the highest from 2009 to 2014, reaching 43.24%. The total storage of forest AGC increased from 1.36×10^7^ Mg C in 1989 to 6.16×10^7^ Mg C in 2019, an increase of 352.94%. This shows that the trend of change in the total storage of forest AGC is consistent with the trend of change in forest AGC density, showing a positive correlation. From 1989 to 2019, the total amount of forest AGC in Lishui City showed a continuous growth trend. Among them, the change in forest AGC was relatively large and the growth was relatively fast during the ten years from 2004 to 2014. The increase in forest AGC was less rapid between 1989 to 2004 and 2014 to 2019.

**Figure 9:**
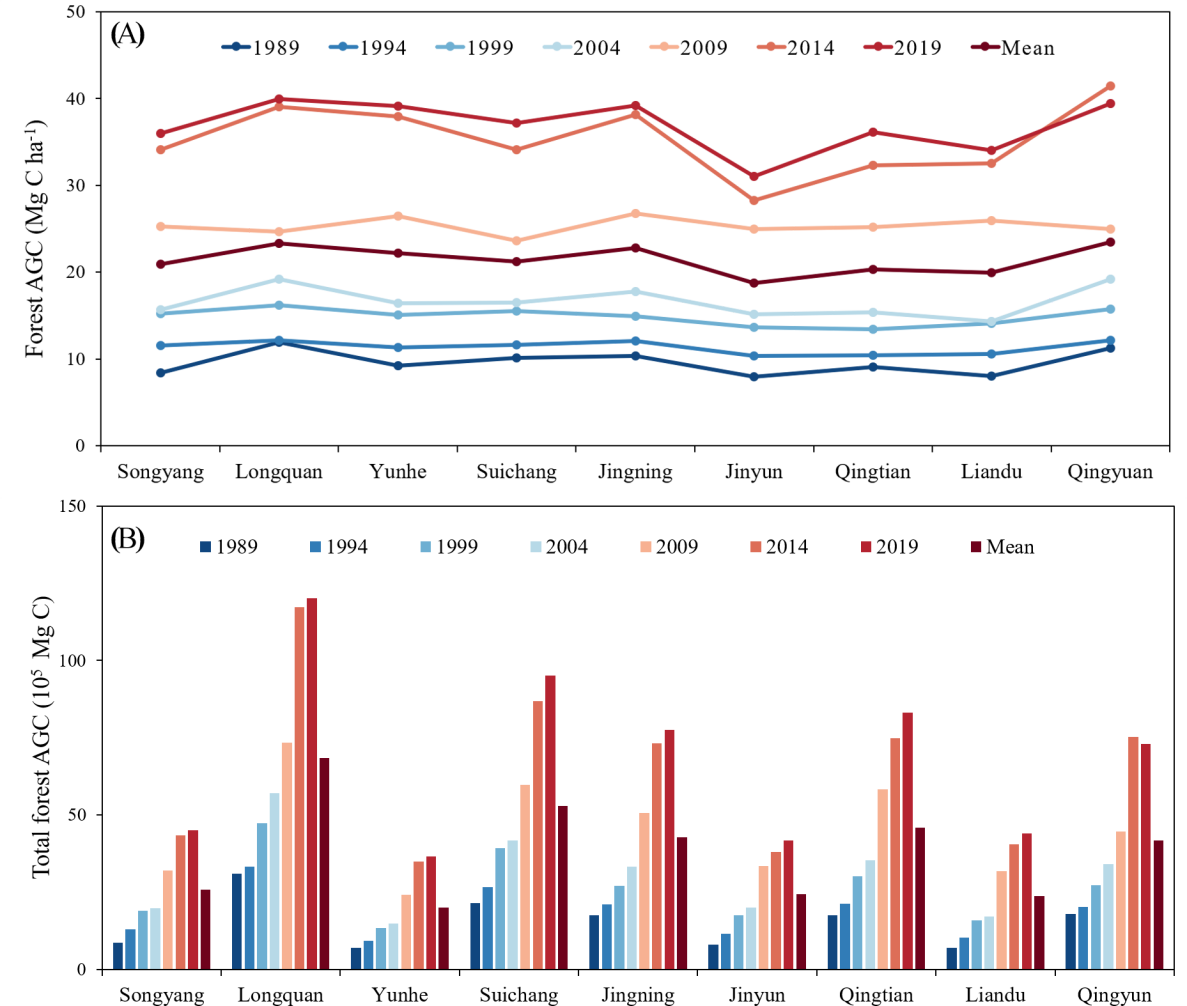
**(A)** Variations in forest AGC across various regions over different years, **(B)** cumulative forest AGC in different regions over various years.

**Figure 9** illustrates the yearly variations in forest AGC and the overall forest AGC storage across nine sectors: Songyang, Longquan, Yunhe, Suichang, Jingning, Jinyun, Qingtian, Liandu, and Qingyuan. Over time, the forest AGC in each division has increased year by year. In all sectors, the forest AGC reached its minimum in 1989 and peaked in 2019. During the study period, among the nine divisions, Longquan had the highest forest AGC, Jinyun had the lowest, and the forest AGC distribution in the other divisions was relatively uniform within the same year.

## 5 Discussion

### 5.1 Variable selection importance analysis

**Figure 10:**
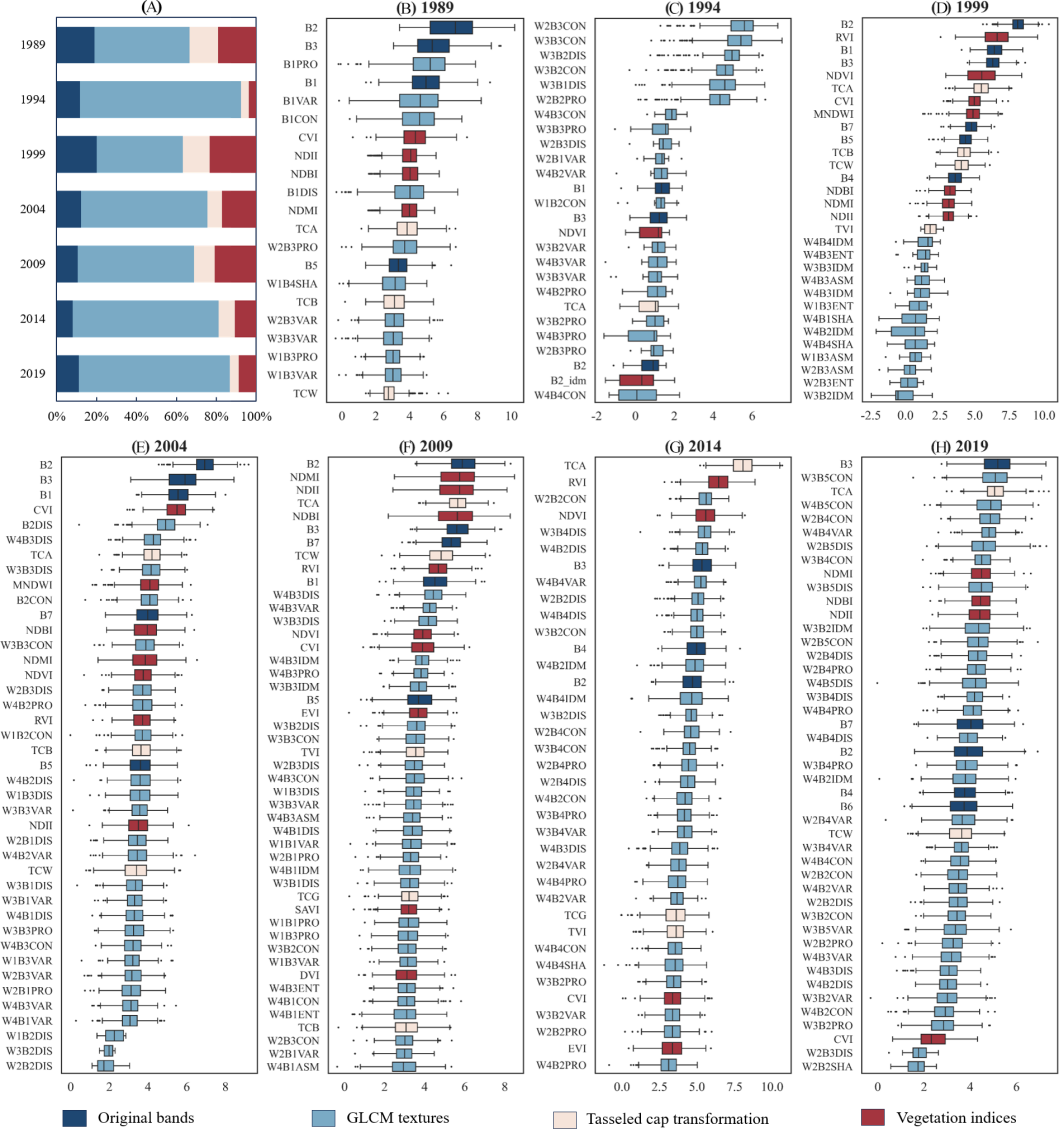
The percentage of the four feature types **(A)** and the result of variable selection on remote sensing data from seven periods using the Boruta variable selection method: 1989, 1994, 1999, 2004, 2009, 2014, and 2019 **(B–H)**.

The construction of the forest AGC model is closely related to the feature variables input into the model. The accuracy of the model is significantly influenced by the input of various feature variables. Therefore, this study uses the Boruta algorithm for variable selection. This eliminates redundant or irrelevant predictive features, thereby improving the performance of the model. The Boruta algorithm is run 500 times in different years to analyze the importance of each feature variable. **Figure 10** shows the results of the feature variable importance ranking. As depicted in **Figure 10**, texture data has the most substantial effect on both the development and precision of the model. In the seven model periods from 1989 to 2019, texture features accounted for 48%, 81%, 43%, 63%, 58%, 73%, and 76% of the feature variables, respectively. In the forest AGC model, the significance of texture information surpasses that of the original bands, vegetation indices, and cap transformation features. Texture information is the most important feature parameter (Shen et al., 2016). The next are the vegetation index and original bands, which account for 14.86% and 13.14% of the total feature variables, respectively. This suggests that surface texture information is the primary element in constructing a forest AGC model, aligning with Zhang’s research findings (Zhang et al., 2019). In addition, the proportions of texture features in the 9 × 9 and 11 × 11 windows are higher. They constitute 28.3% and 35.84% of the overall texture features, respectively.

### 5.2 Model comparison analysis

This study applies three machine learning algorithms to remotely sense and quantitatively estimate the forest AGC in Lishui City from 1989 to 2019. The findings indicate that the CatBoost model outperforms the BPNN and RF models in terms of accuracy. BPNN can establish complex non-linear relationships. Nonetheless, the BPNN model’s capacity to generalize is limited, making it susceptible to overfitting (Yu et al., 2023). Moreover, there are many parameters to adjust in BPNN, and the process of selecting appropriate parameters is complex and lengthy (Ren et al., 2022). While the RF model is capable of processing high-dimensional data and exhibits strong resistance to noise, it tends to overfit during the model construction phase (Georganos and Kalogirou, 2022). CatBoost, as a relatively new machine learning algorithm, has strong robustness to poor data quality and can maintain good performance. This algorithm can also automatically normalize or standardize features (Zhai et al., 2023). Simultaneously, CatBoost incorporates an inherent regularization feature, which can mitigate overfitting to some degree and enhance the model’s predictive performance. Therefore, the overall performance of the CatBoost model is superior to the BPNN and RF models. In terms of forest AGC estimation, the CatBoost algorithm can provide more stable and higher accuracy inversion results. This aligns with the findings from Li’s research (Li H, et al., 2023).

### 5.3 AGC time series variation analysis

Research indicates that from 1989 to 2019, the total forest AGC in Lishui City increased annually. The total forest carbon storage increased from 1.36×10^7^ Mg to 6.61×10^7^ Mg, showing a continuous growth trend. This finding is consistent with the trend identified in Diao’s research (Diao, 2022). However, since Diao’s study centered on better-managed plantation forests, the AGC data obtained from our study are slightly lower than the figures reported by Diao (Diao, 2022). This is because plantation forests typically receive higher levels of management and maintenance. Compared to natural forests, this may lead to superior growing conditions and higher carbon uptake efficiency, resulting in greater carbon storage capacity. After 2000, the forest AGC in Lishui City increased significantly. Firstly, since 1994, Zhejiang Province has implemented projects aimed at cultivating young and middle-aged forests, as well as transforming low-yield and inefficient forests (Huang et al., 2023; Mao et al., 2022). By around 2000, the young forests involved in these projects had matured into middle-aged forests. This resulted in a substantial increase in carbon storage per tree, leading to a notable rise in the overall carbon storage of Lishui City. Secondly, following the invasion of natural secondary pine forests in Zhejiang Province by the pine wood nematode, the province adopted measures to convert coniferous forests into broad-leaved forests (“conifer to broadleaf” conversion) (Diao et al., 2022; Zhang et al., 2007). This gradual transformation significantly increased the proportion of broad-leaved and mixed coniferous-broadleaved forests within the forest landscape, thereby substantially enhancing the forest carbon storage capacity. Thirdly, the plot data used in this study originates from the continuous inventory of forest resources in Zhejiang Province. After 2000, the measured values from the inventory plots showed a significant increase, consequently leading to a corresponding rise in the forest carbon storage calculated through allometric equations. Additionally, the afforestation of high-efficiency and long-term carbon sink forests was promoted to comprehensively protect and restore the mountains, water, forests, fields, lakes, grasses, and sands at the source of the Oujiang River (Gu et al., 2023). Measures such as optimizing forest structure, strengthening the supervision and management of forest land use and harvesting, and controlling pine wilt disease were implemented. These efforts have increased the total forest AGC in Lishui City, expanded the forest area, and improved the ecological benefits of the forest. At the same time, Baishanzu National Geological Park is located in the central and southern parts of Lishui City. The forest AGC in this area is at the forefront of Lishui City and continues to rise. However, due to the impact of natural disasters such as snow disasters and typhoons, carbon storage in some areas of Lishui City has declined annually. In 2019, Typhoon “Lekima” landed in Zhejiang, and the affected forest area in Lishui City reached 610.81 km^2^ of forests (Zhang et al., 2021). Compared with 2014, the forest AGC on the eastern boundary of Lishui City has significantly decreased, and the growth rate of forest AGC has slowed down.

### 5.4 Limitations and prospects

This study provides a methodological reference for accurately monitoring forest carbon stocks. Moreover, the results of this study will offer valuable data support for assessing forest quality through monitoring. There are some limitations in this study. Due to some factors not being fully considered, the study possesses certain limitations. Firstly, the latest Landsat 8 remote sensing image has a resolution of 30 meters and contains 7 original bands. However, as research advances, there is an escalating demand for greater image accuracy and the richness of information that images provide. The problems of insufficient resolution of Landsat remote sensing satellite images and single remote sensing information are gradually emerging (Chen et al., 2021; Puliti et al., 2021). Secondly, the cross-validation grid search method for tuning hyperparameters is not well-suited for continuous hyperparameters. For continuous hyperparameters, grid search typically can only explore at fixed intervals. This can result in the optimal solution being overlooked as it might lie between two grid points. Thirdly, this study employs a single machine learning model, which can lead to scenarios where there are errors in the forest AGC estimation results (Zhai et al., 2023). In estimating forest AGC, the CatBoost model tends to underpredict consistently. Additionally, CatBoost is characterized by its complexity, encompassing numerous parameters, and the training procedure is notably time-consuming (Zhang et al., 2022).

In future research, emphasis should be placed on addressing the aforementioned issues. Subsequent studies could attempt to utilize high-resolution images, such as those from Sentinel-2A (Mallinis et al., 2018). Sentinel-2A data features a resolution of 10 meters, encompassing 13 original bands, particularly including the three red edge bands (Zarco-Tejada et al., 2018). These red edge bands are crucial for estimating forest biomass and AGC, significantly contributing to enhanced data accuracy. Secondly, algorithms such as random search and Bayesian optimization have performed well in hyperparameter tuning, achieving relatively precise results (Park et al., 2023). Simultaneously, in future research, a combination of various model types can be employed to estimate forest AGC. Spatial statistical models such as the Geographically Weighted Regression (GWR) model and Co-Kriging (COK) can effectively reflect spatial heterogeneity (Wang et al., 2021). Integrating different types of models can offset the errors caused by individual models, thereby enhancing the accuracy of the estimation results.

## 6 Conclusion

This study used BPNN, RF, and CatBoost, three machine learning methods, to remotely sense and quantitatively estimate the forest AGC in Lishui City based on Landsat remote sensing images. The results showed:

1. Texture information was a key parameter in constructing the forest AGC model. The Boruta algorithm was used for variable selection. The selection results indicated that texture information had the greatest impact on the construction of the forest AGC model for Lishui City. Among them, the proportions of texture features in the 9×9 and 11×11 windows were the highest.
2. All three machine learning models developed in this research were capable of predicting the forest AGC in Lishui City. Nevertheless, the AGC model built with the CatBoost algorithm demonstrated superior accuracy. When compared to BPNN and RF, the test set accuracy R^2^ of the CatBoost model saw an increase of 12.16% and 2.5% respectively, while the RMSE experienced a reduction of 25.96% and 3.65% respectively.
3. From 1989 to 2019, the forest AGC in Lishui City increased annually. The forest AGC density increased from 10.03 Mg C ha^-1^ to 37.32 Mg C ha^-1^, and the total forest AGC increased from 1.36 × 10^7^ Mg to 6.16 × 10^7^ Mg. This was due to the protection policies and forest transformation policies of Lishui City. However, at the same time, uncontrollable factors such as economic development plans and natural disasters also led to a decrease in forest resources and forest carbon storage in some areas. Therefore, in the future, while accelerating the process of urbanization, there will also be a need to focus on enhancing the conservation of forest resources.

## Data Availability

The data analyzed in this study is subject to the following licenses/restrictions: the data has confidentiality restrictions. Requests to access these datasets should be directed to dhqrs@126.com.
